# A GIS-Based Method for Analysing the Association Between School-Built Environment and Home-School Route Measures with Active Commuting to School in Urban Children and Adolescents

**DOI:** 10.3390/ijerph17072295

**Published:** 2020-03-29

**Authors:** Francisco Sergio Campos-Sánchez, Francisco Javier Abarca-Álvarez, Javier Molina-García, Palma Chillón

**Affiliations:** 1 Department of Urban and Spatial Planning, School of Architecture, University of Granada, 18009 Granada, Spain; fcoabarca@ugr.es; 2 AFIPS research group, Department of Teaching of Musical, Visual and Corporal Expression, University of Valencia, Avda. dels Tarongers, 4, 46022 Valencia, Spain; javier.molina@uv.es; 3 PROFITH ‘PROmoting FITness and Health through Physical Activity’ Research Group, Department of Physical Education and Sport, Faculty of Sport Sciences, University of Granada, 18071 Granada, Spain; pchillon@ugr.es

**Keywords:** active transportation, connectivity, logistic regression, pedestrian route directness, ROC curve, sustainable development goals, walkability

## Abstract

In the current call for a greater human health and well-being as a sustainable development goal, to encourage active commuting to and from school (ACS) seems to be a key factor. Research focusing on the analysis of the association between environmental factors and ACS in children and adolescents has reported limited and inconclusive evidence, so more knowledge is needed about it. The main aim of this study is to examine the association between different built environmental factors of both school neighbourhood and home-school route with ACS of children and adolescents belonging to urban areas. The ACS level was evaluated using a self-reported questionnaire. Built environment variables (i.e., density of residents, street connectivity and mixed land use) within a school catchment area and home-school route characteristics (i.e., distance and pedestrian route directness—PRD) were measured using a geographic information system (GIS) and examined together with ACS levels. Subsequently, the association between environmental factors and ACS was analysed by binary logistic regression. Several cut-off points of the route measures were explored using receiver operating characteristic (ROC) curves. In addition, the PRD was further studied regarding different thresholds. The results showed that 70.5% of the participants were active and there were significant associations between most environmental factors and ACS. Most participants walked to school when routes were short (distance variable in children: OR = 0.980; *p* = 0.038; and adolescents: OR = 0.866; *p* < 0.001) and partially direct (PRD variable in children: OR = 11.334; *p* < 0.001; and adolescents: OR = 3.513; *p* < 0.001), the latter specially for children. Mixed land uses (OR = 2.037; *p* < 0.001) and a high density of street intersections (OR = 1.640; *p* < 0.001) clearly encouraged adolescents walking and slightly discouraged children walking (OR = 0.657, *p* = 0.010; and OR = 0.692, *p* = 0.025, respectively). The assessment of ACS together with the environmental factors using GIS separately for children and adolescents can inform future friendly and sustainable communities.

## 1. Introduction

### 1.1. Active Commuting to/from School (ACS) Contributes to the Sustainable Development Goals (SDGs)

It has been known for decades that a crucial factor in achieving urban sustainability can be reducing society’s dependence on motor vehicles [1]. It is widely recognised that ACS (i.e., non-motorised travel, e.g., walking or cycling) reduces car use, thereby improving health and well-being as suggested by the latest systematic reviews existing in this regard [2,3,4,5]. The authors of these reviews examined many of the studies reporting evidence of the positive impact of ACS on physical activity (*n* = 90), body weight (*n* = 90) and cardiovascular health (*n* = 12). Therefore, ACS is positively related to the Sustainable Development Goals (SDGs) of Agenda 2030 [6] and more specifically, it may contribute to both the SDG 3 ‘good health and well-being’, since practicing active commuting improves human health at every age; and the SDG 11 ‘sustainable cities and communities’ considering that active commuting can reduce the pollution in the cities. The present study focuses mainly on the relationship between the urban built environment and ACS, providing information about how to develop efficient urban policies that raise ACS levels and thus improve health and the environment. This relationship may be connected to the SDG global indicators provided for monitoring both SDGs 3 and SDGs 11 (see global indicator framework adopted by the General Assembly—A/RES/71/313 in https://unstats.un.org/sdgs/indicators/indicators-list/). 

Consequently, a link between ACS and SDGs can be found with several indicators of the SDGs 3, in goals 3.4. (to reduce cardiovascular disease and promote mental health and well-being), 3.6. (to reduce traffic accidents), 3.9. (to reduce air pollution), and with the SDGs 11 in goals 11.6 (improve air quality) and 11.a (to support environmental, social and economic linkages between urban, periurban and rural areas).

### 1.2. ACS Behaviour

The health benefits of active commuting are widely recognized. As evidence of these benefits, the recent systematic review conducted by Larouche et al. (2014) [5] found support for active commuting in improving physical activity levels (49 studies), body weight (39 studies) and cardiovascular fitness (10 studies). Active commuting to school also promotes other benefits such as independent mobility, improved social relationships, mental health and connection to urban and natural environments in children and adolescents [7]. However, it appears that ACS rates are generally decreasing due to the increased use of motorized transport [8,9,10]. Therefore, studying ACS correlates and enforcing them through appropriate policies may help change this behaviour. 

A growing number of studies suggest the existence of multiple factors influencing the ACS of young people on school travels [11]. According to ecological models of health behaviour [12], multiple interaction levels between personal characteristics (i.e., intrapersonal), psychosocial (e.g., interpersonal modelling and social support) and environmental factors (e.g., neighbourhood and home, school and workplace environments) and policies (e.g., health care and transport policies, zoning codes and traffic demand) determine active living and physical activity. Specifically, correlates of ACS were studied including demographic, family, school and social factors [13]. Considering these factors, observed rates of ACS were higher in children than in adolescents [14], and in those of lower versus (vs.) higher socio-economic status [15,16]. Likewise, among others, parental concerns regarding personal and traffic safety [17,18], as well as safety perceptions of walking routes [19] also influence how young people travel to/from school.

According to the systematic reviews from D’Haese et al. (2015) [20] and Wong et al. (2011) [21], many studies assessed environmental correlates of ACS in children/adolescents using objective methods such as Geographical Information Systems (GIS). Moreover, most studies analysed separately the home or school-built environment or the home-school route, but very few studied several built environments (e.g., [22]). In addition, since the built environment is highly contextual and differs in every country, more research is needed in Spain since there are few studies about the built environment and ACS [23,24,25]. For example, the study of Rodríguez-López et al. (2017) [26], that used the same Spanish urban sample as in the current study, only studied the distance from home to school but they did not include any home or school neighbourhood variables. New research integrating built environment and route to/from school factors analyses would help to identify specific urban interventions of ACS for each population subgroup including children and adolescents.

### 1.3. Environmental Factors that May Influence ACS of Children and Adolescents

The built environment may influence ACS across three general dimensions (i.e., density, diversity and design), which may be evaluated around the school, home or home-school routes [27]. For example, among others, (a) density has to do with compactness encouraging non-motorized travel to school and shorter school trips; (b) diversity may mean having mixed land use destinations; and (c) design features, including pedestrian/cycling infrastructure and gridded street patters, may increase destinations active accessibility. Other objective environmental measures based on street design may be topography, traffic safety and aesthetics [21,28]. 

The influence of these environmental dimensions and measures also depends on whether children or adolescents are studied, in addition to the type of built environment. For example, Huertas-Delgado et al. (2017) [18] reported barriers evidence of Spanish parents on traffic volume for children and distance to school for adolescents. Dangerous intersections and crime were reported as barriers for both age subgroups. Bringolf-Isler et al. [29] found positive associations between main street crossings along home-school routes and non-active commuting in children, while Timperio et al. (2006) [30] did not observe an association between the presence of busy roads (as barriers) along the home-school route and ACS. The latter authors also found a negative association between steep slope along the route to school and ACS in children but not in adolescents [30]. Mitra et al. (2010) [31] observed that the density of industrial (manufacturing/trade) and office employment had a high and negative association with ACS to/from school in adolescents, while retail and service employment had no association with ACS. In addition, there is evidence of more ACS trips for children attending schools located in lower socio-economic status (SES) neighbourhoods [23,32], and more obesity and body fat for adolescents from lower-SES home neighbourhoods [24]. However, Ikeda et al. (2018a) [32] found that ACS was negatively associated with school SES for youths. In addition, a short distance to school was found to be the strongest positive correlate with ACS in most cases [33,34,35].

#### 1.3.1. Built Environment Measures

Traditionally in the field of urban design, transport and planning, three of the most relevant factors of the built environment commonly used as indicators of walkability are (i) density of residents, (ii) street connectivity and (iii) mixed land uses [16,21,23,32,36,37,38]. It may mean that the presence of people, a dense grid of well-connected streets, and the functional complexity of land uses can support walkability [38]. However, research focusing on children and adolescents reported limited evidence and non-conclusive associations of these three built environment factors with ACS [21]. For example, Ikeda et al. (2018a) [32] found association between increased street connectivity around schools and ACS for both children and youth. However, they also found a negative relationship between dwelling density and distance to school with ACS, this distance being the strongest predictor of ACS [32]. Larsen et al. (2009) [39] reported significant positive associations of land-use mix in the school-neighbourhood with ACS in children, but no relationship between them was found in the home-neighbourhood. McDonald (2007b) [40] reported no association between land-use mix and ACS in children. Moreover, Molina-García and Queralt (2017a) [23] found non-significant associations between school-neighbourhood walkability (as an index of residential density, land-use mix and street connectivity based on GIS data) and ACS. In contrast, ACS behaviour was more frequent in lower-walkable home-neighbourhoods among Spanish adolescents [24]. In addition, Queralt and Molina-García (2019) [41] observed a positive association between street connectivity and independent mobility to different destinations (not specifically to school) in Spanish adolescents’ home-neighbourhoods.

From a scale perspective, these three built environment measures (i.e., density of residents, street connectivity and mixed land uses) are considered macro-scale features as they are urban morphology characteristics. However, there are also micro-scale features consisting of small environmental details that may also affect walkability [42] such as traffic calming features, aesthetics attributes, parking areas existence and pavement quality [21]. This study focused on macro-scalar attributes of the school-neighbourhood. 

#### 1.3.2. Home-School Route Measures

Street connectivity is considered by planning to be a key factor in urban design. From an urban network perspective, the more the urban fabric looks like a dense and continuous grid, the higher the connectivity [43,44]. Low connectivity means that the origin-destination route through the street network is less direct and therefore the distance to be covered is increased. Origin-destination long distance discourages ACS and hence physical activity, which affects the scope of SDGs. Low connectivity may be due, for example, to low street network density, unlinks within the urban grid, the large size and length of blocks and the existence of cul-de-sacs, among others [43].

Two street connectivity measures were addressed in this study. The density of street intersections as a measure of the school-built environment and, in addition, the pedestrian route directness (PRD) as a performance measure of the home-school route of each child/adolescent (i.e., direct vs. indirect routes). In addition, a previous study indicated that the PRD variable is considered to be the best predictor of ACS among other connectivity variables such as street network density, connected node ratio, intersection density and link-node ratio [43]. 

The PRD measure was used in Portland (USA) to fix the maximum length of urban blocks, with a limit value to consider the routes as direct of PRD = 1.5 [44]. Taking into account the study of Randall and Baetz (2001) [1], values for PRD = 1.4–1.5 mean neighbourhood-grid street patterns and relatively small blocks. In contrast, values for PRD = 1.63–1.88 show irregular streets and cul-de-sacs existence. The INDEX model sets values for PRD = 1.2–1.5 as direct routes while values for PRD = 1.6–1.8 indicate indirect routes [45]. Timperio et al. (2006) [30] classified the pedestrian routes of children to school as direct when they were <1.6 and indirect when they were ≥1.6. For these authors, negative correlates of ACS included parental perceptions of children, no lights or crossings to use, a busy road barrier, a steep incline route to school and a good connectivity [30]. In addition, until the beginning of the 21st century the PRD measure had not yet been used by local governments [44].

The greater the number of shorter and a priori more direct routes (i.e., less home-school distance) the higher the ACS level. Apart from route measures, ACS may also depend on other factors such as traffic level [17,18,19], which will be discussed regarding these measures. Dill’s research (2004) [43] gathered international evidence on the use of the PRD measure to show how direct/indirect are certain origin-destination routes. Nevertheless, how direct/indirect home-school routes are for Spanish children/adolescents in urban areas have not yet been studied. Knowing the association between ACS and how direct/indirect the home-school routes are as well as other environmental factors (i.e., density of residents, density of street intersections and mixed land uses) may help urban policy and planning decision-makers on the built environment encouraging walkability to/from school.

The main aim of the work was to examine the association between different environmental factors of both school-neighbourhood and home-school route with ACS in Spanish children and adolescents of urban areas. In addition, the work aimed to identify threshold data for route variables, i.e., the overall threshold distance for participants’ ACS, as well as the overall threshold PRD and separately by participants’ age group. This study can be useful for urban policy and planning for the development of built environment interventions improving ACS levels, thus helping the scope of related SDGs.

## 2. Materials and Methods 

### 2.1. Study Sample and Design

The participants’ data came from a cross-sectional study conducted in November 2012. The participants were primary school children (7–11 years old) and secondary school adolescents (12–18 years old) initially belonging to 26 schools of cities with >20,000 inhabitants of the southeast of Spain distributed in the provinces of Granada, Almeria and Murcia. These schools were recruited as a sample of convenience. Initially, 4777 students agreed to participate in the study by completing a questionnaire. It included questions such as personal data (e.g., age, family postal address, gender and school) and how participants commuted to/from school during the study week (i.e., the question from which the number of times a week participants walked to/from school was known). The questionnaire was approved by the Ethics Committee on Human Research of the University of Granada (Spain). All schools involved in the study were informed of the purpose of the questionnaire. Each school informed the participants and their parents in order to obtain their acceptance and written consent. 

### 2.2. Active Mode of Commuting to/from School

The participants completed a self-reported questionnaire helped by the teachers. The questions on the mode of transport to the school were set with the support of the systematic review on this respect by Herrador-Colmenero et al. (2014) [46] and were previously validated by the study of Chillón et al. (2017) [47]. The most important question asked regarding this work was about the mode of weekly travel (5 days, 2 possible times per day) to/from school. This question identified the number of times (0–10) that participants went to/from school and in which mode (i.e., active vs. non-active). The answer options were (a) walking or cycling (both coded as active commuting), although cycling was not included in the sample because of the low sample, or (b) using car, motorbike or bus (coded as non-active commuting). The sample of the study was very biased to the right. In other words, many participants reported reaching the maximum value of ACS (ACS = 10) in their travels to/from school from home. In the dichotomous recoding process, active participants (1) were considered to be those who reported an ACS value of [4–10], and non-active participants (0) those who reported an ACS value of [0–3], according to the study by Chillón et al. (2014) [48]. In addition, socio-demographic data were reported in the questionnaire such as the participants’ age group and gender, among others.

### 2.3. Built Environmental Variables

All the built environmental variables (i.e., school-built environment measures and home-school route measures) were calculated using a spatial analysis with the software QGIS V.3.4. The input data came from the spatial analysis of (i) the school-built environment, as well as (ii) the home-school route. Both data were collected at the participant level to create environmental exposure variables to be used in further analyses.

**Figure 1:**
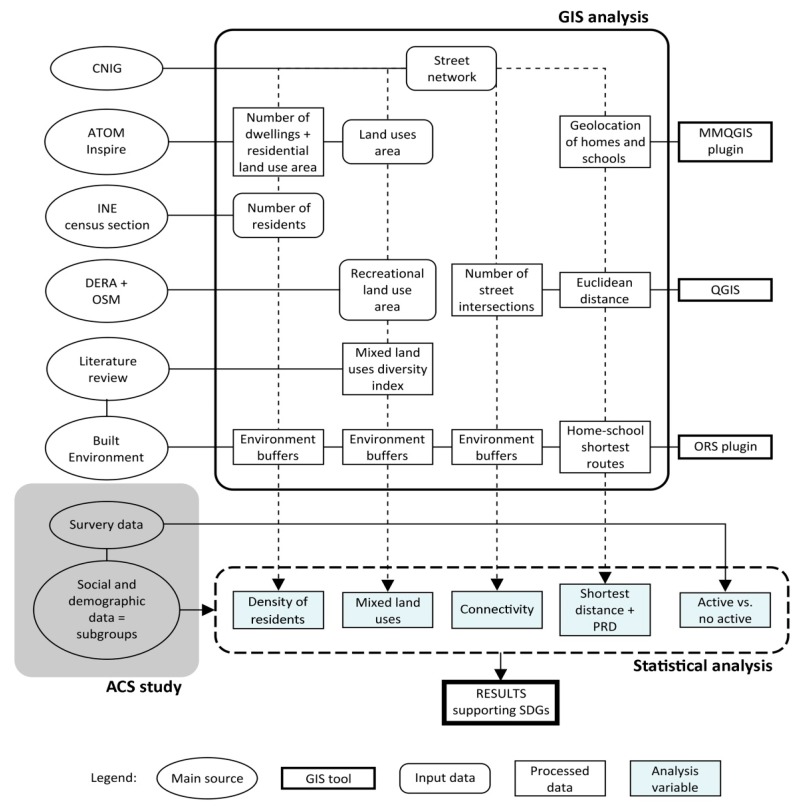
Method workflow. Abbreviations: CNIG: National Centre for Geographic Information. ATOM Inspire: National cadastral service. INE: National Statistics Institute. DERA: Reference Spatial Data of Andalusia (Spain). OSM: Open Street Map. PRD: Pedestrian route directness. ORS: Open Route Service. SDGs: Sustainable Development Goals. GIS: Geographic Information System. ACS: Active commuting to school. Additional note: The literature review was a source of processed data since it allowed finding previous studies useful to define both the analysis variable of mixed land use and the built environment catchment area.

The urban street network vector data as a base map for developing the subsequent spatial analysis was obtained from the road network digital information of the CNIG (National Centre for Geographic Information, as translated from its Spanish acronym) (date of the GIS data source: 2017). Figure 1 shows a workflow scheme of the method.

#### 2.3.1. School-Built Environment

The built environment of each school (i.e., buffer in term of spatial analysis) was defined as the area between the school and the threshold distance covered in every direction through the street network. This distance is what a pedestrian (child or adolescents in this case) is willing to walk. Several built environments were identified depending on the threshold distance that the participants are willing to cover in a predominantly active way. In order to identify the different environments (*n* = 2), the threshold distances for the children/adolescents from urban areas collected in the research of Rodríguez-López et al. (2017) [26] were used as follows: 1250 m for children and 1350 m for adolescents.

The density of residents consists on the ratio of the number of residents to land area (area values given in hectares) of the school-neighbourhood buffer (i.e., residents variable). The density of street intersections as a connectivity measure consists on the number of street intersections to land area of the school-neighbourhood buffer (i.e., intersections variable). These methods for measuring the density of residents and the density of street intersections were used before by other authors [21,29,49]. The mixed land uses (i.e., the integration level of land uses in a given area) were measured by a mixed-use diversity index (i.e., mixed uses variable). It captures how evenly the square footage of several urban land uses (i.e., residential, industrial, retail, office, public service and recreational) is distributed within each school-built environment. Therefore, it could be said that it is an indicator of urban dynamism or vitality in terms of diversity and functional urban complexity. 

The number of residents and the urban land use areas of each built environment were obtained from the ATOM Inspire national cadastral service (date of the GIS data source: 2018). This was done by means of disaggregation and aggregation operations based on available information on the number of dwellings per building, types of land use and resident population data. The latter came from the available information in the census section of the National Statistics Institute (INE) (date of the GIS data source: 2014). Although there are several methods for analysing the diversity of mixed land uses, e.g., [21,50,51], here the mixed-use diversity index (processed as a z-value, i.e., a normalised value) was obtained by the procedure from International Physical Activity and the Environment Network (IPEN; www.ipenproject.org) methodology, based on the method of Frank et al. (2005) [52]. The DERA (Reference Spatial Data of Andalusia; date of the GIS data source: 2017) as well as vector data from Open Street Map (OSM; date of the GIS data source: 2017) collaborative database were used to measure the recreational area (i.e., parks, gardens, playgrounds, sport fields and other open spaces) within this index. The higher this mixed-use diversity index value is the more mixed the land use, which a priori is positive to increase the ACS levels.

#### 2.3.2. Home-School Route

The distance between home (identified by family postal address of each participant) and school was calculated using the shortest distance on the street network between both (i.e., distance variable), as previous studies did (e.g., [22,30]). The Pedestrian Route Directness refers to the ratio between the shortest distance of the home-school route through the street network and the home-school straight-line distance (i.e., the Euclidean distance) (i.e., PRD variable).

The geolocation of homes and schools (i.e., the spatial georeferencing of their postal addresses) was carried out using MMQGIS, a set of Python plugins developed by Michael Minn. Previously, the participants’ family postal addresses, originally compiled in an Excel sheet, were exported to CSV format. This plugin used Google Maps web service as well as an application programming interface (API) key that enabled the geolocation process and allowed to examine the home-school routes. The shortest routes between children/adolescents’ homes and schools, as well as the buffers (i.e., isochrones in terms of time) used in the spatial analysis of the different variables, were obtained using the Open Route Service (ORS) plugin supported by the Heidelberg Institute for Geoinformation Technology (HeiGIT). The geoprocessing operations performed using both plugins were implemented in the GIS used.

### 2.4. Statistical Analysis

The statistical analysis was developed using SPSS 23 software (SPSS Inc., Chicago, IL, USA). All of the above-mentioned environmental variables (as independent or predictive variables) were continuous and not normal (bilateral asymptotic significance <0.05 after Kolmogorov-Smirnov test). Their influence on ACS was studied using binary logistic regression (BLR). This is a type of statistical analysis commonly used in the association between the built environment and ACS (e.g., [26,34]). In BLR, the probabilities described by a single dependent variable are modelled based on several predictive variables using a logistic function. The Intro method was used. It consists of a non-automatic procedure by which all variables are entered in a single step. 

The dependent variable (ACS variable) was recoded as a dichotomous categorical variable, i.e., active or walkers (4-10 active travels/week) vs. non-active, passive or motorised commuters (0-3 active travels/week). In addition, due to the low sample size of the school-built environment variables (n < 30) (i.e., residents, intersections and mixed uses variables), these variables were also categorised into dummy variables using the median as the cut-off point. The route predictors (i.e., distance and PRD) remained as numerical variables. The statistical analysis was developed in several steps as follows. 

(1) A first BLR (model 1) was carried out for all participants by adding every environmental predictive variable (i.e., residents, intersections, mixed uses, distance and PRD). The age group variable (children vs. adolescents) was additionally added to the model as a categorical predictor in order to check its statistical significance and thus its applicability as a sample adjust variable for further analysis. In addition, the regression coefficients (i.e., the odds ratio—OR—as the ratio of the odds of ACS in the presence of exposure or independent variables) of the predictive variables obtained by including into the model the age group variable and without including it were compared to check for confounding effects. 

(2) The multicollinearity of these predictive variables was checked (i) by requesting correlation matrix in the BLR (i.e., no collinearity when correlation coefficients between predictors < 0.80 and the standard errors < 2.0 [53]); and (ii) using a multiple linear regression model (MLR) and requesting the collinearity diagnostics as recommended by SPSS manual (i.e., no collinearity when the variance inflator factor (VIF) is higher than 1.0 and the condition index <20 [54]).

(3) Considering the results of the previous step, the association between the environmental predictors and the ACS variable was examined using a second BLR (model 2) separately for children and adolescents.

(4) The threshold PRD of ACS for all participants and separately for children/adolescents, and the threshold distance of ACS for all participants were calculated using a receiver operating characteristics (ROC) curve analysis, which have already been studied previously (e.g., [1,26]). ROC curve analysis was widely used in several scientific fields when the evaluation of discrimination performance was of interest in the research [55]. The larger the area under the curve is (values between 0 and 1), the more discriminatory the test and the better the analysis model. Cut-off points between active vs. non-active participants for these variables were obtained using the Youden Index or J, where J = sensitivity + specificity – 1. This is the vertical distance between the ROC curve and the diagonal line [56]. The cut-off point for the threshold values mentioned above was obtained from the maximum value of J (i.e., maximum vertical distance). The ROC curve model was considered valid when the area under the curve ≥0.5 and the value 0.5 is outside the 95% CI (confidence interval). Otherwise the model was considered only exploratory. Once the significance variables were determined, an additional bivariate correlation analysis was carried out in order to discard multicollinearity between some of them. 

(5) In addition, the cross-table analysis allowed us to know the participants ACS level considering threshold PRD for all participants and for active vs. non-active participants. In addition, cross-table analysis made it possible to know the participants ACS level according to different ranges of PRD identifying to what extent the routes were direct or indirect [1,43] for different participants subgroups. 

## 3. Results

Participants were excluded from the analyses if they (i) did not complete the questionnaire regarding the number of total active travels to the school (*n* = 720); (ii) reported commuting to/from school by bike (*n* = 16); (iii) did not provide or did not write down correctly the information of their family postal address, which would ensure its correct georeferencing and therefore the correct calculation of the home-school distance; and (iv) were lost or null cases (*n* = 1073). The final sample for the analyses was 2968 participants from 24 schools (8 primary schools for children, 14 secondary schools for adolescents and 2 primary-secondary schools for children and adolescents). This sample did not filter some possible cases of young people who mistakenly wrote down a different address than the family postal address, which in some isolated cases could be very far from the urban areas of the study.

Table 1 shows the participant characteristics according to socio-demographic variables (e.g., age and gender) and mode of travel to/from school (i.e., active vs. non-active). There was a higher percentage of active participants than non-active, more adolescents than children and slightly more men than women. There were more active children than non-active, and more active adolescent than non-active. The percentage of active adolescents over the total adolescent participants was higher than the percentage of active children over the total children participants. There were the same number of non-active women and men. However, the percentage of active men over the total male participants was slightly higher than the percentage of active women over the total female participants. Additionally, Table 2 shows the descriptive statistics of the predictive variables. 

The results of the first BLR (model 1) showed that all predictive variables were found to be significant (*p* ≤ 0.05), except for the residents variable (*p* = 0.1). Subsequently, the statistical significance of the age group variable was verified (*p* < 0.001) (goodness of fit was 72.9% for all participants; cut-off value for the classification of cases *p* = 0.50, i.e., cases with predicted values above the cut-off value for classification have the event or result modelled, while cases with predicted values below the cut-off value do not have the event or result, with *p* being a cut-off value between 0.01 and 0.99), this variable being accepted as an adjust variable for the BLR of model 2. In addition, the difference between the ORs of the predictive variables obtained by BLR (model 1) including the age group variable and without including it was found to be about or less than 10% [57]. 

The correlation matrix in BLR of model 1 showed correlation coefficients between predictors <0.80 and standard errors were <2.0. Despite this, the collinearity diagnostics from a MLR were requested to contrast them with each other. Regarding the latter, some of the collinearity results were considered improvable (i.e., residents variable VIF = 2.157, mixed uses variable VIF = 1.764 and PRD variable condition index = 15.71). A second round of MLR collinearity diagnostics was requested without including the residents variable since it was found to be non-significant according to BLR (model 1) results. The collinearity results improved and showed this time that every predictive variable VIF was slightly higher than 1.0 (e.g., mixed uses variable VIF = 1.073) and the condition index for every predictive variable was <15. Therefore, residents variable was removed from the second BLR (model 2). 

Table 3 shows the association between ACS and environmental predictors separately by age group using BLR of model 2 (goodness of fit was 67.7% for children and 77.4% for adolescents; cut-off value *p* = 0.50). The school-built environment and route variables (i.e., intersections, mixed uses, distance and PRD) were statistically significant for both children and adolescents. The direction of the intersections and mixed uses variables was negative for children and positive for adolescents (i.e., the higher the density of intersections and the more mixed the land uses, the more ACS for adolescents and the less ACS for children). The direction of the distance variable was always negative (i.e., the shorter the distance the more ACS for all participants). The direction of the PRD variable was positive for all participants (i.e., the less direct the route, the more ACS). Finally, the strength of the PRD variable for children should be noted (OR = 11.33). 

**Figure 2:**
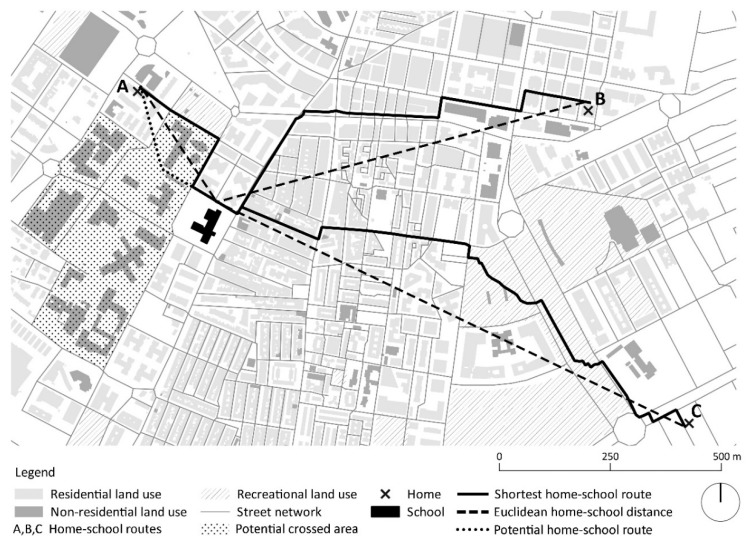
Example of spatial analysis of the pedestrian route directness (PDR) variable in a school-built environment.

Figure 2 shows an example of the spatial analysis carried out for the PRD measure in one of the school-built environment of the study (Granada, Spain). Considering PRD value as the ratio between the home-school route shortest distance through the street network (continuous line) and the home-school straight-line distance (dashed line), both home-school routes B (PRD = 1.221) and C (PRD = 1.192) were more direct than home-school route A (PRD = 1.304). In the case of route B this is due to the fact that the urban layout through which this crossed route looked like a grid, being therefore its connectivity high. Route C additionally crossed a public park, making the route to/from the school even more direct. In contrast, route A was shorter than the others but not more direct since pedestrian had to border a wide non-crossable area of no-residential land use to reach the school. In addition, the figure shows a potential home-school route (dotted line) through this area (in case it was possible to cross it through, which will be discussed later) that would allow a more direct route than route A (i.e., PRD < 1.304).

**Figure 3:**
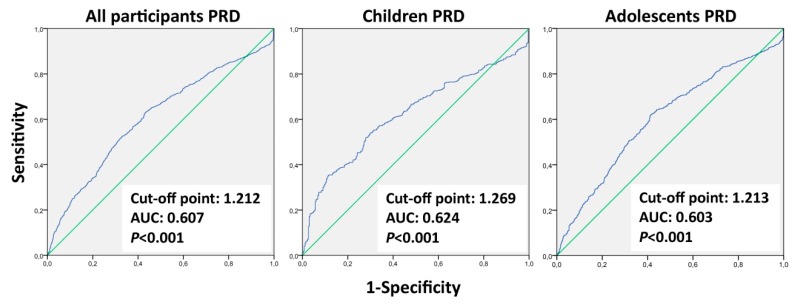
Receiver operating characteristic (ROC) curves of ACS by PRD measure separately by age groups. Note: PRD = Pedestrian route directness; AUC = Area under the curve.

Figure 3 shows the results of the ROC curve analysis of ACS by PRD for all participants and separately for children and adolescents. The threshold PRD for all participants was 1.212 (J max. = 0.178) (i.e., below this value non-active participants predominated, and the active ones were above it). The threshold PRD separately by age group was for children = 1.269 (J max. = 0.242) and for adolescents = 1.213 (J max. = 0.207). All cases were significant. The ROC curve of ACS by PRD should be considered valid but only exploratory (0.5 < AUC < 0.75) in all cases. In addition, the ROC curve analysis of ACS by distance variable for all participants (not shown) was a valid model (AUC = 0.900). The threshold distance (i.e., cut-off point) for active vs. non-active participants was 1.262 km (*p* < 0.001; J max. = 0.677), prevailing active participants below this value and non-active participants were above it.

Table 4 shows the cross-table analysis of ACS by PRD variable. ACS was associated with PRD ranges in order to know the number/percentage of active and non-active participants who commuted to/from school in each range. This knowledge allowed us to know the PRD range in which the highest number of active and non-active participants commuted to/from school. Therefore, it may be useful to guide built environment and urban design policies in order to achieve the optimal PRD for promoting walkability to/from school and reducing car use. 

The analysis included both the cut-off point of threshold PRD = 1.212 and the cut-off point of −PRD = 1.5 as key cut-off points of the association between ACS and PRD measure. The former was included to explore the non-active (PRD ≤ 1.212) vs. active (PRD > 1.212) participants’ behaviour, and the latter to explore direct (PRD ≤ 1.5) vs. indirect (PRD > 1.5) routes to/from school according to the study of Dill (2004) [33]. A total of 70.5% of participants reported active travel to/from school, with a high percentage of them (from 63.3% to 89.2%) taking direct routes (1.212 < PRD ≤ 1.5). Only 10.8% of active participants took indirect routes (PRD > 1.5) to/from school. Instead, 29.5% of participants reported non-active commuting to/from school, with most of them taking direct routes (PRD ≤ 1.5; 96.2%) and highly direct routes (PRD ≤ 1.212; 56.4%). 

In addition, on the one hand, most of the participants’ active behaviour (62.6%) was explained with ranges [Min.–1.212] and (1.212–1.30]. However, most of non-active participants (56.6%) already predominated with range [Min.–1.212]. On the other hand, if we take into account the ratio between active and non-active participants in each range (not showed in the table), the behaviour of those active ones was better explained as the routes to/from school tended to be indirect (i.e., PRD ranges close to the value of 1.50 or more). In contrast, the non-active behaviour was better explained when routes were direct, i.e., PRD ranges below the interval (1.50–Max.]. 

## 4. Discussion

The main findings of this study reported that there were more active than non-active commuters to/from school. There were associations of ACS with most of the school and route environmental measures (i.e., density of intersections, mixed land uses, distance and pedestrian route directness) in children and adolescents. In addition, a threshold PRD value (PRD = 1.212) was found for active (values above the threshold PRD) vs. non-active participants (values below the threshold PRD) of the sample and separately for children (PRD = 1.269) and adolescents (PRD = 1.213). In addition, most active and non-active participants preferred direct routes to/from school, mainly the non-active ones. Furthermore, in the case of most active participants, the routes to/from school were about 30% longer than their straight-line routes (i.e., Euclidean routes). However, in the case of most non-active participants, the difference between these routes was only about 20%.

### 4.1. Home-School Route Measures 

Distance is the strongest predictor of ACS. In the current study, it showed a high significant association with ACS and most participants were more likely to actively travel to/from school when the route was short (threshold distance = 1.262 km). This agrees with many earlier studies (e.g., [34,35]). The previous study, where the similar urban sample participated, reported a threshold distance for children (875 m) and adolescents (1350 m) [26], which is also consistent with the research of Hume et al. (2009) [58] and Panter et al. (2013) [59]. These authors observed that children living less than 1 km from school were more likely to ACS. However, there is also empirical evidence that this distance is often greater for children [33] and adolescents [60]. Regardless of this, the distance of about 1 km is also a reference distance for ACS analysis used in a wide range of studies (e.g., [22,36,41]). 

As a general trend, the lower the PRD value the more ACS in all cases, i.e., the higher the directness of the route the more ACS in all cases. In addition, all participants clearly preferred direct rather than indirect routes, especially the non-active ones. Almost two thirds of active travels happened when routes deviated 30% from the straight-line route to/from school (i.e., the Euclidean route), and more than three quarters of them as routes deviated 40%. Beyond 50% deviation (i.e., indirect routes), the walking trend increased slightly, but the number of participants decreased greatly overall. The higher rates of ACS as routes tending to be not too direct are consistent with previous similar studies (e.g., [30,61]). The PRD measure was analysed individually for each participant rather than each school-built environment, being significant for both children and adolescents. Therefore, PRD measure was confirmed as the best predictor of connectivity in our study, as reported by previous research (e.g., [43]).

### 4.2. School-Built Environment and Home-School Route Correlates of ACS for Children and Adolescents

Children were more likely to actively travel to/from school when routes (i) tended to be calm (mixed uses variable had negative sign; OR = 0.657); (ii) were less connected (intersections variable had negative sing; OR = 0.692); (iii) were short distance (distance OR = 0.980; threshold distance = 1.262 km); and (iv) were almost one third longer than straight-line routes (PRD OR = 11.334; threshold PRD = 1.269 for non-active vs. active). In contrast, adolescents were more likely to actively travel to/from school when routes (i) had more street intersections (OR = 1.640); (ii) were dynamic and functional (mixed uses had positive sign; OR = 2.037); (iii) were short distance (OR = 0.866; threshold distance = 1.262 km); and (iii) were about 20% longer than straight-line routes (PRD OR = 3.513; threshold PRD = 1.212).

There was a positive association between ACS in adolescents and a high density of street intersections, which was in line with what was reported by previous research (e.g., [62]). However, no association was found between ACS in children and a high density of street intersections by other studies (e.g., [49]). Moreover, a negative association was found between a high density of street intersections and ACS in children, which is consistent with the relevant study of Larsen et al. (2012) in London (Ontario) [63]. Instead, McDonald (2007b) [40] indicated that a high density of residents was associated with ACS when school trips were in long distance. However, it should be noted that in the case study active travels to/from school predominated in short distances, which may explain why this time the density of residents was not a significant factor. However, a high number of residents of a given urban area may be related to an improvement in the safety of this area as confirmed decades ago by the famous study of Jacobs (1961) [64] and supported by the more recent research of Gehl (2010) [65], among others. The mixed land uses measure also correlated with ACS in line with other studies (e.g., [38,39]) but differently according to age subgroups. It seems that active adolescents clearly passed through complex functional urban areas to/from school, while children tended slightly to avoid them as indicated earlier by other studies (e.g., [63]). 

It must be noted that this study used GIS-estimated shortest routes rather than GPS-derived or child-mapped routes. Previous studies showed that GPS-derived/child-mapped routes (and associated environmental exposure) were different form GIS-estimated shorted routes [66,67]. Furthermore, the choice of street networks influences GIS-estimated school routes [68]. These two aspects should be taken into account when interpreting the results of the present study. In addition, the school-built environment analysis using GIS and wide street network buffers helped in obtaining significant environmental correlations, which was previously evidenced by Van Loon et al. (2014) [69]. 

The finding regarding the children preference for walking along routes to/from school tending to be less direct than for adolescents is, a priori, scarcely intuitive and differs from what was reported by some authors such as Braza et al. (2004) [49] and Saelens et al. (2003) [38], although the latter referred mainly to adults. It should be also noted that only about one tenth of all active participants went to/from school using clearly indirect routes. It is therefore clear that this aspect requires further study. However, these results are consistent with what was observed by other authors such as Panter et al. (2010) [22] and Timperio et al. (2006) [30] and may reflect differences in behaviour among children and adolescents.

It seems that children active travels to/from school were influenced by other environmental factors such as traffic safety, among others, which may move them away from more direct routes (i.e., usually with high levels of traffic, air pollution and noise). This influence is in line with other previous studies (e.g., [17,18,19,63]). In addition, the recent study of Egli et al. (2019) [70] found that children who actively travel to school are aware of traffic safety, air pollution and prefer commuting with friends and/or family members to socialize. These are aspects that may have less influence in active adolescents or adults. According to this, here the threshold PRD for non-active vs. active commuting is lower in adolescents than in children (i.e., fewer deviations from the straight-line route to/from school for adolescents, i.e., more direct routes to/from school for adolescents than for children), this aspect being also in line with the study of Babey et al. (2009) [71]. 

Apart from that, neighbourhoods with poorly connected street networks and some cul-de-sacs (i.e., high values of PRD and a low density value of street intersections) may provide less traffic-exposed routes and thus safer, which may be preferable for children. In contrast, well-connected grid street networks (i.e., low values of PRD, i.e., more direct routes) favour traffic [68]. In line with this, the PRD factor of ACS is strongly present in school travels of children living in urban areas (PRD OR = 11.344) as observed. Large cities are usually provided with busy roads. It would be interesting for future research to study this correlate in rural areas where roads are not as busy, but at the same time the safety features of pedestrian streets (e.g., wide pavements, safe crossings, road signs and protection elements) are less common than in urban areas [72].

The probabilities of adolescents to actively travel to/from school depended mainly on functional dynamism level of the urban area (i.e., the mixed uses variable was significant and with a positive sign; OR = 2.037). These odds also increased when routes were short-distance (distance OR = 0.866) and only about 20% longer than the straight-line ones (PRD OR = 3.513; threshold PRD = 1.213). In other words, it seems that adolescents walked to/from school along the main streets (i.e., more direct, dynamic and well-connected than the secondary streets, i.e., with more diverse and better combined urban land uses, and with a high density of street intersections). It seems that in this case, the influence of the PRD measure had more to do with the school-built environment and/or main street dynamism than with road safety or noise, bearing in mind that adolescents are not as sensitive to traffic volume as children, the latter already indicated in previous studies (e.g., [63]. The study of this relationship in rural areas would be of interest for future research, due to the fact that the integration level of land uses in these areas is generally lower (i.e., they are commonly not highly dynamic areas) than in urban areas. Taking into account the odds ratio values (OR) of PRD (Table 3), it seems that the pedestrian safety factor against traffic/safety/noise for children, in the terms described above, is even more influential than the urban dynamism factor for adolescents, if the comparison is possible.

Finally, the potential of large areas of certain non-residential public land use areas (e.g., educational, recreational and even health land use) to be passed through by pedestrian routes (including home-school routes) was illustrated in Figure 2. This type of routes may support strong but friendly institutional identities as well as spatial differentiations and improved street life [73]. In addition, such shortcuts passing through built environments with restricted and/or light traffic can lead to more direct home-school routes which may increase pedestrian safety and walkability. This is in line, for example, with the recent study of Campos-Sánchez et al. (2019) [74] on accessibility to urban green areas and their network use value measure (i.e., multiple and diverse use) for people. The latter increased when non-residential land use areas showed potential to be passed through by pedestrian routes. Other urban design/policy measures to reduce the length of home-school routes and increase their directness may include limiting the block length and size (or allowing shortcuts through them) and avoiding cul-de-sacs [43,44]. In addition, avoiding single land use areas, increasing residential density and increasing the number and improving the design of pedestrian safety features against traffic may support ACS high levels for children and/or adolescents [1,19,63,72].

### 4.3. Strengths and Limitations of the Study

The strengths of the study included the use of a validated measure of ACS as well as the analysis of environmental factors using GIS-based measures. In addition, the analysis of the built environment factors together with route measures under an integrated approach may help to detail the influence level of these factors and measures on young people, which may be useful for urban policy and planning decision-making. Furthermore, this study carried out in the Spanish context added geographic and cultural diversity to prior research. 

However, a limitation of this work was designing the database regardless of the input data rules of the MMQGIS plugin, which was used for the geolocation of the participants’ family postal addresses. In fact, information other than the family postal address that caused errors during the geolocation process was sometimes noted in the Excel database. As a result of this, a lot of participants data were lost and thus removed from the sample (*n* = 1073). The sample finally used in this study was wide (*n* = 2968) and this data loss did not prevent its development. 

Another limitation was to analyse only three (most important) macro-scale built environment measures of ACS, but not other important influencing factors such as safety, aesthetics or topography. The micro-scale features of the built environment were also not studied, and there is also evidence of their influence in ACS [42]. Moreover, the discussion regarding threshold values of the PRD measure has to be considered exploratory, due to the flatness of the ROC curve regarding the main diagonal of the schemes. In addition, the statistical analyses were not clustered by school.

In addition, the school-built environment and the home-school route were studied, but the home and the route built environments were not. The latter can also influence how young people commute to/from school [22], so this lack is another limitation of the study. Finally, the findings from this study were based only on walkers (excluding cyclists) as a limitation. 

## 5. Conclusions

In this work, there were more active than non-active commuters to/from school, and adolescents were more active than children. From a functional urban perspective, active adolescents were associated with routes passing through dynamic and functional school-built environments and/or along main streets, unlike children. The latter were associated with routes that deviated from the shortest routes between home and school more than adolescents. In addition, adolescents were more active travelling to/from school when the street network within the school-built environment was more connected, unlike children. Instead, for both children and adolescents ACS increased when home-school routes were shorter and direct. This proved that environmental correlates of ACS should be evidenced by population subgroups in order to detail the results and recommendations derived from them. Likewise, it was verified that distance and PRD are suitable measures for school-travel assessment, also confirming that PRD is the best connectivity predictor in this case. The main strengths of the study had to do with the use of valid measures in assessing ACS, as well as adding geographic and cultural diversity to prior research of the case study. The main limitations of the study related to the limited number and type of measures and built environments analysed, and the database design for home addresses geocoding. In addition, the study of environmental factors of influence may support urban policy and planning decision-making increasing ACS levels in urban children and adolescents. This knowledge may improve health and well-being in cities contributing to achieve the SDGs.

## Figures and Tables

**Table 1:** Sample cases descriptive frequencies separately by participants’ subgroups.

Sample Cases	All *n* = 2968 (100.0%)	Active *n* = 2091 (70.5%)	Non-Active *n* = 877 (29.5%)
Children	826 (100.0%)	561 (67.9%)	265 (32.1%)
Adolescents	2142 (100.0%)	1530 (71.4%)	612 (28.6%)
Male	1508 (100.0%)	1070 (71.0%)	438 (29.0%)
Female	1460 (100.0%)	1022 (70.0%)	438 (30.0%)

**Table 2:** Environmental variables descriptive statistics separately by participants’ subgroups.

Participants’ Subgroups	Statistics	Environmental Variables					
		ACS (nº of Active Travels/Week)	Residents (nº of Residents/Buffer)	Intersections (nº of Street Crossings/Buffer)	Mixed Uses (Index)	Distance (km)	PRD (Index)
All	Mean	6.53	158.83	3.74	0.00	2.93	1.28
	Median	10.00	158.91	3.75	-0.06	0.80	1.24
	SD	4.36	57.31	1.24	0.87	9.80	0.23
	Min	0.00	66.51	1.44	-1.43	0.01	0.03
	Max	10.00	271.61	6.63	2.00	112.61	4.57
Active	Mean	9.18	156.12	3.68	0.03	1.55	1.30
	Median	10.00	158.91	3.75	-0.06	0.62	1.26
	SD	1.73	59.46	1.31	0.85	7.86	0.25
	Min	4.00	66.51	1.44	-1.43	0.01	0.03
	Max	10.00	271.61	6.63	2.00	111.44	4.57
Non-active	Mean	0.20	165.28	3.90	-0.08	6.23	1.24
	Median	0.00	170.66	3.74	-0.08	2.99	1.20
	SD	0.61	51.29	1.03	0.89	12.74	0.17
	Min	0.00	66.51	1.44	-1.43	0.04	0.55
	Max	3.00	271.61	6.63	2.00	112.61	3.45
Children	Mean	6.12	156.84	3.78	0.30	2.16	1.30
	Median	9.00	136.17	3.76	0.30	0.69	1.25
	SD	4.35	64.39	1.49	1.07	7.86	0.22
	Min	0.00	66.51	1.44	-1.28	0.03	0.93
	Max	10.00	271.61	6.63	2.00	85.74	3.13
Adolescents	Mean	6.68	159.59	3.73	-0.12	3.23	1.28
	Median	10.00	158.91	3.74	-0.06	0.86	1.23
	SD	4.36	54.33	1.13	0.74	10.44	0.24
	Min	0.00	67.46	1.56	-1.43	0.01	0.03
	Max	10.00	234.50	6.54	1.19	112.61	4.57

Notes: Mean = The sum of the values of a data set divided by the number of the values; SD = Standard deviation; Median = The value separating the higher half from the lower half of a data sample; ACS = Active commuting to and from school; PRD = Pedestrian route directness; Min = Minimum value; Max = Maximum value.

**Table 3:** Predictors of ACS separately for children and adolescents using binary logistic regression (BLR) (model 2).

Age Group	Statistics	Environmental Variables			
		Intersections	Mixed Uses	Distance	PRD
**Children**	**B**	−0.369	−0.420	−0.021	2.428
	**SE**	0.165	0.164	0.010	0.485
	P	0.025	0.010	0.038	<0.001
	**OR**	0.692	0.657	0.980	11.334
	**95% CI**	0.500–0.955	0.477–0.906	0.961–0.999	4.384–29.303
**Adolescents**	**B**	0.495	0.711	−0.144	1.257
	**SE**	0.114	0.116	0.016	0.298
	P	<0.001	<0.001	<0.001	<0.001
	**OR**	1.640	2.037	0.866	3.513
	**95% CI**	1.312–2.050	1.624–2.555	0.839–0.894	1.960–6.297

Notes: The sign of B indicates the direction of the relationship between the dependent variable and the predictive variables; OR (odds ratio) indicates the strength of the relationship between the dependent variable and the predictive variables. SE = Standard error; CI = Confidence interval (95%) for the odds ratio estimation; P = Statistical significance. Variable are statistically significant when *p* ≤0.05. PRD = Pedestrian route directness.

**Table 4:** Cross-table analysis of ACS by PRD ranges.

ACS	PRD Ranges					
	All	[Min.–1.212]	(1.212–1.30]	(1.30–1.40]	(1.40–1.50]	(1.50–Max.]
**All**	2955 (100%)	1263 (100%)	717 (100%)	506 (100%)	211 (100%)	258 (100%)
**Active**	2081 (100%)	768 (36.9%)	534 (25.7%)	378 (18.2%)	176 (8.4%)	225 (10.8%)
**Non-active**	874 (100%)	495 (56.6%)	183 (20.9%)	128 (14.7%)	35 (4.0%)	33 (3.8%)

Notes: Lost cases = 13; Pearson’s Chi-square test < 0.001; Max. = Maximum value; Min. = Minimum value; PRD = Pedestrian route directness; ACS = Active commuting to/from school
